# Aspirin Suppressed PD-L1 Expression through Suppressing KAT5 and Subsequently Inhibited PD-1 and PD-L1 Signaling to Attenuate OC Development

**DOI:** 10.1155/2022/4664651

**Published:** 2022-03-29

**Authors:** Xiyun Xiao, Saitian Zeng, Yanying Li, Lingling Li, Jiyan Zhang

**Affiliations:** Department of Gynaecology, Cangzhou Central Hospital, Cangzhou, Hebei Province, China

## Abstract

Ovarian cancer (OC) is a frequently occurred malignancy with high incidence and poor survival worldwide. In recent years, immune checkpoint inhibition that targets PD-1/PD-L1 axis has become an efficient and popular therapy for cancers. Aspirin (ASP), an anti-inflammatory drug, exhibits a wide spectrum of biological functions including anticancer property. However, the role of ASP treatment in ovarian cancer treatment remains unclear. In this work, we explored the role of ASP in modulating PD-L1 signaling during OC development. Notably, *in vitro* experiments showed that ASP treatment caused repressed proliferation of OC cells. The results from *in vivo* xenograft model suggested suppressed tumor growth and tumor weight under ASP treatment. ASP treatment also caused downregulated PD-L1 and Ki-67 levels in mice tumors. Moreover, the IFN-*γ*-caused PD-L1 accumulation was inhibited by ASP treatment. The administration of ASP decreased the expression of PD-L1 of OC cells in a coculture system with activated T cell or unstimulated PBMCs, along with decreased expression of PD-1 by activated T cells. ASP reversed PD-L1 expression caused by coculture with activated T cells and abolished the suppressed T cells activation and proliferation. Analysis on molecular mechanisms revealed that KAT5 bonded to the promoter region of PD-L1 and upregulated its expression via enhancing histone H3 lysine 27 acetylation (H3K27ac), whereas ASP downregulated KAT5 expression and blocked this phenomenon. Moreover, ASP enhanced the effect of antiPD-L1 therapy in the *in vivo* tumor model. Hence, we proposed that ASP decreased expression of PD-L1 protein via inhibiting the epigenetic regulation by KAT5 and suppressed the PD-1/PD-L1 signaling to attenuate tumor growth. ASP may be a promising adjuvant in OC immunotherapy.

## 1. Introduction

Ovarian cancer (OC) is the primary cause of gynecologic cancer-related death globally [1]. Though the therapeutic approaches of OC have been advanced, the current treatment is limited because of the occurrence of drug resistance [2, 3]. Immune therapies, especially the immune checkpoint inhibitors (ICIs) that target the immune-suppressive proteins programmed death 1 (PD-1) and its ligand programmed death-ligand 1 (PD-L1) and the cytotoxic T lymphocyte-associated antigen-4 (CTLA-4), have been widely applied in clinical therapy of solid tumors in recent years and achieved quite good outcomes [4–6]. Accumulating evidences suggested that targeting PD-1/PD-L1 axis facilitates the activation and antitumor effects of T cells [7, 8]. On the other hand, comparison with traditional radiotherapy and chemotherapy suggested that blocking the PD-1 and PD-L1 signaling remarkably enhanced clinical response and overall survival of patients, along with less side effects [9]. Hence, exploring safe and efficient approaches that block the PD-1 and PD-L1 signaling is promising and imperative for treatment of OC.

Aspirin (ASP), as an anti-inflammatory drug, has been widely used in treatment of various diseases in clinics [10]. Studies have proved that ASP modulates multiple cellular biological processes, including the cell proliferation, migration, and apoptosis [11]. Recently, increasing number of studies revealed that ASP is an effective agent in treatment of OC [12]. However, the role of ASP on PD-1/PD-L1 axis and immunotherapy is still obscure. Lysine acetyltransferase 5 (KAT5) is acetylases that epigenetically modulates gene expression and is able to induce acetylation of both nonhistone and histone proteins [13, 14]. KAT5 is one of the most crucial lysine acetyltransferases, which has recently been suggested as a potential therapeutic target for cancers, such as colon and breast cancers [13, 15, 16]. Moreover, it has been reported that ASP metabolite salicylate represses KAT5 and MUC1 to inhibit epithelial to mesenchymal transition in prostate cancer cells [17], but the effect of ASP on KAT5 and the association of KAT5 with PD-L1 remain unclear.

In present research, we evaluated the functions of ASP in immunotherapy of OC and determined reduced level of PD-L1 in OC cells and decreased level of PD-1 in activated T cells. Further discussion on molecular mechanisms demonstrated that ASP epigenetically modulated the expression of PD-L1 in OC cells. Our findings provided novel evidence for the application of ASP in the immunotherapy of OC.

## 2. Materials and Methods

### 2.1. Cell Lines and Treatment

Human OC cellsSKOV-3 and TOV-21G and murine OC cell ID8 were obtained from American Type Culture Collection (ATCC, USA). All cells were maintained in DMEM (Hyclone, USA) that contains 10% fetal bovine serum (FBS; Hyclone, USA) at a 37°C incubator filled with 5% CO_2_. For coculture, the cancer cells were seeded in the lower chamber of Transwell plates (Costar, USA), and T cells were placed in the top chambers. The culture medium was collected and centrifuged to obtain conditioned medium. The conditioned medium was mixed with same volume of fresh medium for use.

### 2.2. Cell Transfection

Small hairpin RNAs (shRNAs) that targets PD-L1 (shPD-L1) and KAT5 (shKAT5) were synthesized by GenePharma (China). For cell transfection, Lipofectamine 2000 (Invitrogen, USA) was adopted following the manufacturer's instruction.

### 2.3. Cell Viability and Proliferation

Cell viability and proliferation were assessed by using cell counting kit-8 (CCK-8; Beyotime, China), colony formation, and 5-ethynyl-2′-deoxyuridine (EdU) assay. For CCK-8 assay, OC cells were placed in a 96-well plate (3,000 cells/well) and cultured for indicated time. Then CCK-8 reagent was added to incubate for another 2 h. The absorbance values were measured at 450 nm in a microplate reader (Thermo, USA).

For colony formation assay, the cells were suspended as single cells and seeded in a 12-well plate (500 cells/well) and cultured for 14 days. Next, the colonies were dyed with crystal violet (Sigma, USA) and captured by a digital camera (Nikon, Japan).

To conduct EDU assay, cells were fixed with 4% paraformaldehyde and permeabilized with Triton X-100 after treatment, followed by incubation with 50 *μ*M EDU reagent (Beyotime, China). The nuclei were stained with Hoechst 33342. The positive staining was captured under a fluorescence microscope (Leica, Germany).

### 2.4. T Cell Activation

To obtain T cells, we collected the peripheral blood mononuclear cells (PBMCs) from healthy donors using the Ficoll method and extracted the T cells using a Pan-T cell isolation kit (Invitrogen, USA) in line with the manufacturer's protocol. Next, the T cells were primed by using a T Cell Activation Kit (Invitrogen, USA). Briefly, T cells were incubated with MACS Beads (Invitrogen, USA) preconjugated with antiCD8, antiCD2, and antiCD3 antibodies and then loaded in FACS (BD Biosciences, USA) for isolation.

### 2.5. Flow Cytometry

The determination of cell surface biomarkers was realized by flow cytometry. In brief, cells were suspended in PBS solution with 2% FBS and incubated with fluorescence-conjugated antibodies against CD4, CD8, PD-1, and PD-L1 for 30 minutes in dark. Next, the samples were washed with PBS and loaded in the FACS (BD Biosciences, Germany).

### 2.6. Xenograft Tumor Model

Five-week-old male C57BL/6 J mice were purchased from the Charles River Laboratory (USA). Murine ID8 cells (10^6^ cells/100 *μ*L PBS) were subcutaneously inoculated to the left fat pad of each mouse. The mice were randomly divided into experimental groups when tumor size reached 100 mm^3^. For PD-L1 blockade, mice were intraperitoneally injected with anti-PD-L1 antibody (Abcam, USA) at a dose of 5 mg/kg for three times within one week after grouping. The ASP was administrated through gavage every day at a dose of 20 mg/kg. Mice in the control group were injected with Ig-G antibody and saline as control. The size of tumors and body weight were recorded every other two days. All animal experiments were authorized and conducted following the guideline of Animal Ethic Committee of our hospital.

### 2.7. Immunohistochemical (IHC) Staining

The tumor tissues were isolated, fixed, dehydrated, embedded by paraffin, and cut into 5-*μ*m-thick slices. The samples were processes with antigen retrieval and 3% hydrogen peroxide, blocked with 5% BSA, and hatched with primary anti-Ki-67 antibody overnight at 4°C. Then, samples were stained with 3,3-diaminobenzidine (DAB) and captured under microscope (Leica, Germany).

### 2.8. Quantitative Real-Time PCR (qRT-PCR) Assay

RNA was isolated from tumors and cells by using a TRIzol reagent (Thermo, USA). Transcription to cDNA was conducted using First-Strand Synthesis Kit (Transgen, China). Gene levels were quantified using the SYBR Green manner following the 2^−*ΔΔ*Ct^ method. Relative quantification was conducted using *β*-Actin as the internal control.

### 2.9. Chromatin Immunoprecipitation (ChIP) Assay

ChIP assay was performed to evaluate the interaction between proteins and PD-L1 promoter region. In brief, OC cells were cross-linked in 1% formaldehyde, lysed, and sonicated to obtain chromatin fragments with length around 500 bp. Next, the fragments were incubated with antiKAT5, antiRNA polymerase II, antiH3K27Ac, or anti-IgG as control at 4°C overnight in rotation and then hatched with Dynabeads (Sigma, USA) at 4°C for another one hour. The samples were eluted with proteinase K (Sigma, USA) and quantified with qRT-PCR assay.

### 2.10. Statistical Analysis

The data in this work were shown as mean ± SD of at least three independent experiments. GraphPad Prism software was adopted for statistical analysis. The comparison between two or more groups was conducted using Student *t*-test or one-way ANOVA. *P* < 0.05 was regarded as statistical significance.

## 3. Results

### 3.1. Aspirin Suppresses OC Cell Proliferation

**Figure 1:**
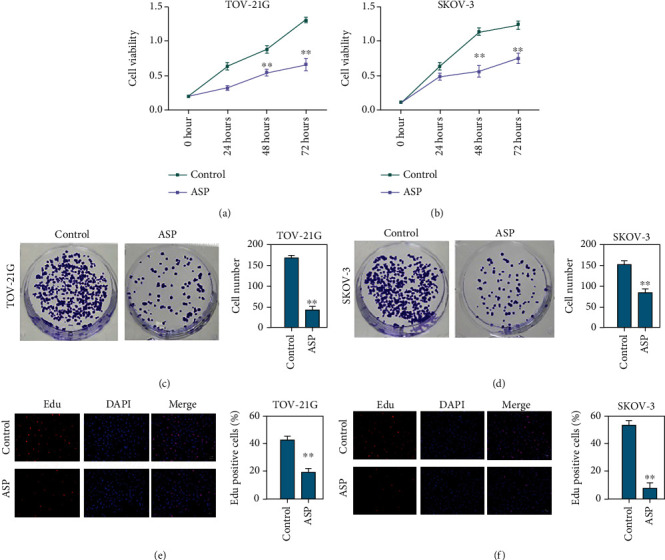
Aspirin suppresses OC cell proliferation. The OC cells were treated with aspirin: (a and b) the CCK-8 assay to check the cell viability; (c and d) colony formation assay was performed to check OC cell proliferation; (e and f) EDU assay to assess cell proliferation. ∗∗*P* < 0.01.

Initially, we assessed the effect of aspirin on OC cells. Results from CCK-8 indicated that aspirin notably suppressed the viabilities of TOV-21G and SKOV-3 cells (Figures 1(a) and 1(b)). The colony formation ability of TOV-21G and SKOV-3 cells was suppressed by aspirin (Figures 1(c) and 1(d)). Moreover, the EDU-positive staining of TOV-21G and SKOV-3 cells was decreased by aspirin (Figures 1(e) and 1(f)).

### 3.2. Aspirin Inhibits OC Cell Tumorigenesis

**Figure 2:**
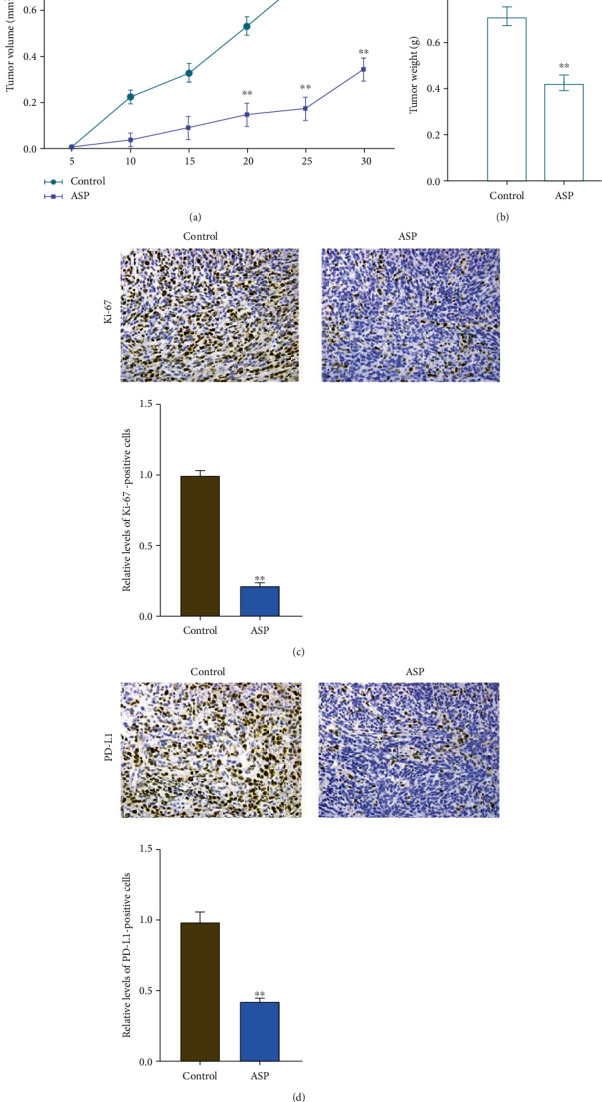
Aspirin suppresses *in vivo* OC cell growth. The TOV-21G cells were injected into nude mice, followed by treatment with aspirin. Then, tumor growth (a) and tumor weight (b) were measured. (c and d) IHC to check the levels of Ki-67 and PD-L1. *N* = 5, ∗∗*P* < 0.01.

We next explored the effects of aspirin on *in vivo* OC model. Tumorigenesis in nude mice showed that aspirin treatment attenuated the tumor growth and the tumor weight in the nude mouse xenograft model (Figures 2(a) and 2(b)). Moreover, results from IHC staining indicated reduced expression of Ki-67 under the administration of aspirin, along with reduced PD-L1 level (Figures 2(c) and 2(d)).

### 3.3. Aspirin Decreases IFN-*γ*-Stimulated PD-L1 Expression in OC Cells

**Figure 3:**
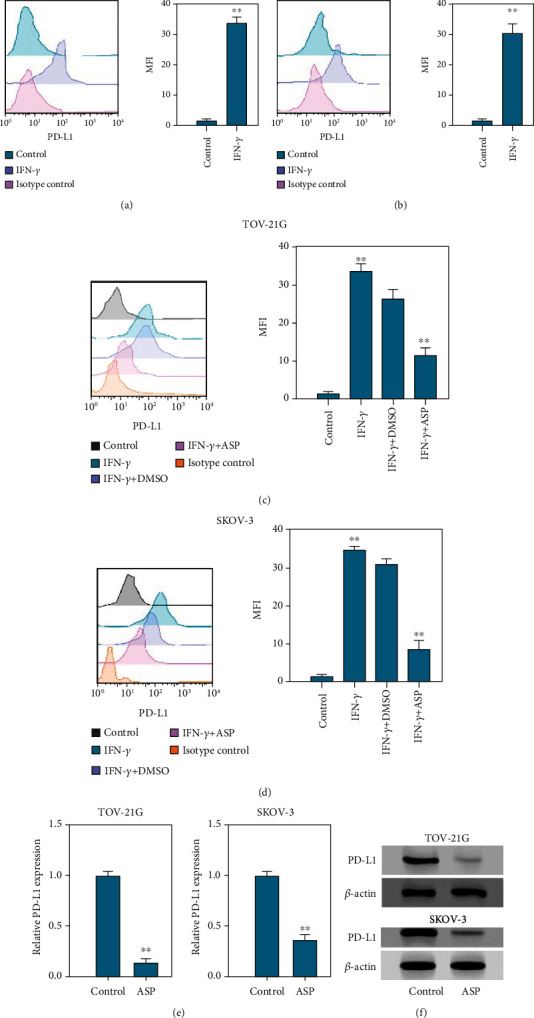
Aspirin suppresses IFN-*γ*-stimulated PD-L1 expression in OC cells. (a and b) TOV-21G and SKOV-3 cells were stimulated with IFN-*γ* at a dose of 200 ng/mL, and then level of PD-L1 was measured by using FACS assay. (c and d) The level of PD-L1 in TOV-21G and SKOV-3 cells under treatment with IFN-*γ* alone or together with aspirin was assessed by FACS. (e and f) The RNA and protein levels of PD-L1 in TOV-21G and SKOV-3 cells were assessed by qPCR assay and western blotting. ∗∗*P* < 0.01.

It is well-known that IFN-*γ* is a classic stimulator for PD-L1 expression. Here, we explored the function of aspirin in IFN-*γ*-induced PD-L1 enrichment. Flow cytometry analysis revealed that IFN-*γ* treatment notably stimulated the level of PD-L1 in OC cells (Figures 3(a) and 3(b)). Nevertheless, flow cytometry analysis demonstrated that the cotreatment with aspirin significantly suppressed the IFN-*γ*-stimulated PD-L1 level in TOV-21G and SKOV-3 cells (Figures 3(c) and 3(d)). Moreover, qPCR and western blot analysis determined that aspirin treatment directly decreased PD-L1 level in OC cells (Figures 3(e) and 3(f)).

### 3.4. Aspirin Promotes T Cells Proliferation and Activation to Inhibit OC Cell Survival

**Figure 4:**
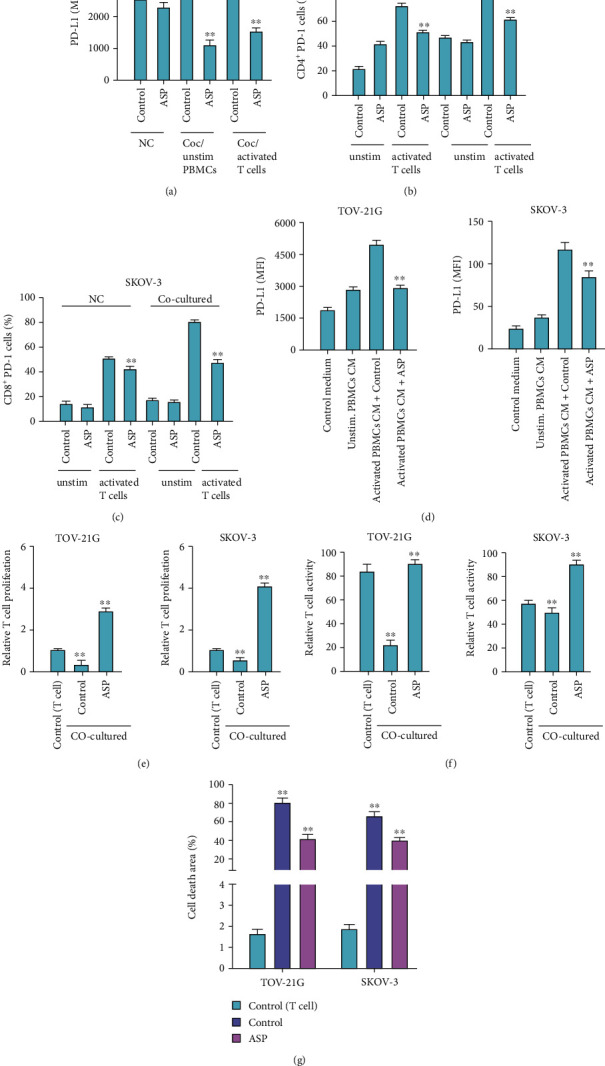
Aspirin stimulates T cells proliferation and activation to inhibit OC cell survival. (a–e) OC cells were cocultured with unstimulated or activated T cells in Transwell. (a) The level of PD-L1 in cells was checked by FACS. (b and c) Flow cytometry to check the portion of CD8^+^ PD-1^+^ and CD4^+^ PD-1^+^ T cell. (d) Level of PD-L1 in TOV-21G and SKOV-3 cells incubated with conditioned medium collected from unstimulated or activated PBMCs. (e) CCK-8 assay to detect proliferation of T cells in the coculture system. (f) Flow cytometry to determine the portion of CD8+ T cells in the coculture system. (g) Flow cytometry to measure T cell apoptosis. ∗∗*P* < 0.01.

Subsequently, we explored the effects of aspirin on T cell function via using a cocultured system, that composed by activated or naive T cells in top chambers and OC cells in the bottom. Flow cytometry analysis revealed that treatment with aspirin suppressed PD-L1 level in TOV-21G cells that cocultured with activated T cell or naive PBMCs (Figure 4(a)). Besides, flow cytometry analysis demonstrated that aspirin reduced PD-1 level in activated T cells that cocultured with OC cells (Figures 4(b) and 4(c)). To further explore whether T cells modulate OC cell behaviors via releasing cytokines, we collected the conditioned medium of PBMCs to stimulate cancer cells, and flow cytometry analysis determined elevated expression of PD-L1 (Figure 4(d)). Moreover, CCK-8 assays showed that aspirin treatment reversed the conditioned medium-stimulated PD-L1 expression (Figure 4(d)). Coculture with OC cells significantly inhibited the activation and proliferation of T cell, whereas aspirin treatment abolished the inhibition (Figures 4(e) and 4(f)). Consistently, flow cytometry analysis indicated that aspirin treatment significantly alleviated cell death of T cells in the coculture system (Figure 4(g)).

### 3.5. Aspirin Epigenetically Regulate PD-L1 Expression in OC Cells via Regulating KAT5

**Figure 5:**
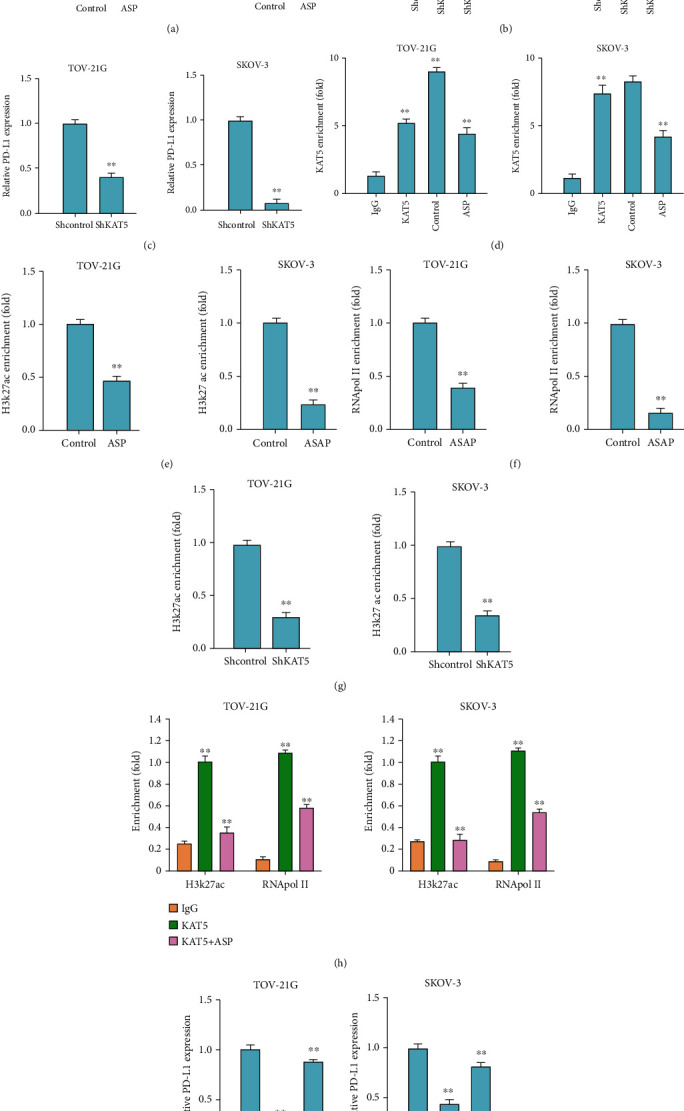
Aspirin decreases PD-L1 expression in OC cells via targeting KAT5. (a–c) The levels of KAT5 and PD-L1 in OC cells was checked by qPCR assay. (d–f) OC cells were treated with aspirin, and then ChIP assay was conducted to check enrichment of (d) KAT5, (e) H3K27ac, and (f) RNA polymerase II on promoter region of PD-L1. (g) ChIP assay to assess the enrichment of H3K27ac on promoter of PD-L1 in OC cells transfected with shKAT5. (h) The interaction of KAT5 with H3K27ac and RNA polymerase II on promoter region of PD-L1 was measured by ChIP-re-ChIP analysis. (i) QRT-PCR assay to check PD-L1 level in OC cells treated with aspirin alone or with KAT5 overexpressing plasmid. ∗∗*P* < 0.01.

We next discussed the molecular mechanism of aspirin repressing PD-L1 level in OC cells. The qPCR analysis showed that aspirin treatment led to reduced KAT5 level in TOV-21G and SKOV-3 cells (Figure 5(a)). The qPCR analysis revealed that KAT5 depletion by shRNAs significantly suppressed the PD-L1 expression in OC cells (Figures 5(b) and 5(c)). Results from ChIP assay demonstrated that KAT5 was able to be recruited to the PD-L1 promoter region, whereas aspirin suppressed this enrichment (Figure 5(d)). And the enrichment of H3K27ac and RNA polymerase II on the promoter of PD-L1 was also impaired by aspirin (Figures 5(e) and 5(f)). Noteworthy, ChIP assay indicated that the knockdown of KAT5 suppressed the recruitment of H3K27ac and the PD-L1 level (Figure 5(g)). Moreover, ChIP-re-ChIP analysis showed the interaction of KAT5 with H3K27ac and RNA polymerase II on the PD-L1 promoter and demonstrated that ASP could inhibit the interaction (Figure 5(h)). On the other hand, qPCR analysis showed that overexpression of KAT5 could recover the suppressed PD-L1 expression under aspirin treatment in TOV-21G and SKOV-3 cells (Figure 5(i)).

### 3.6. Aspirin Improves AntiPD-L1 Immunotherapy in In Vivo OC Model

**Figure 6:**
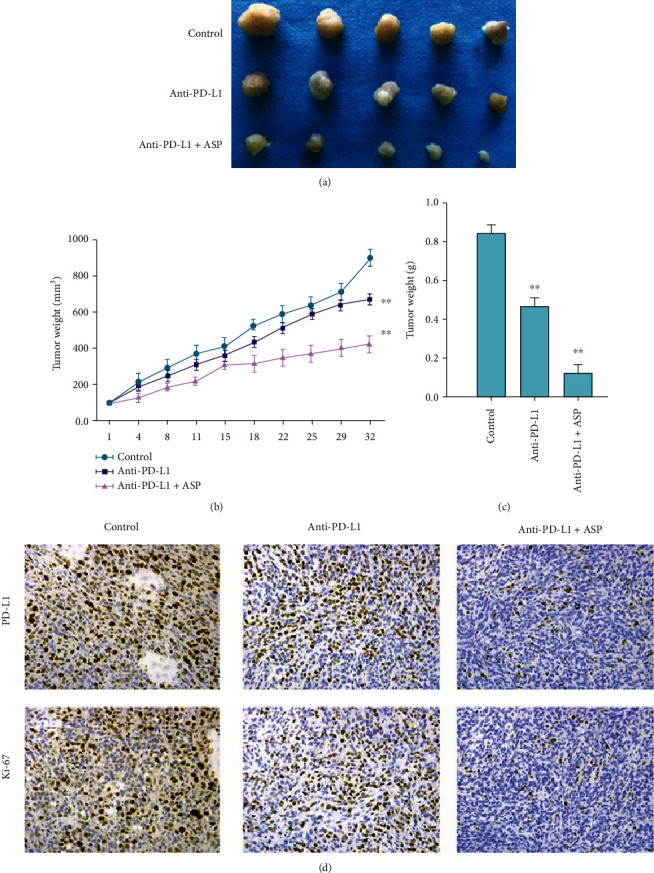
Aspirin improves the effect of antiPD-L1 therapy against OC *in vivo*. Murine OC ID8 cells were injected to C57BL/6 mice. The mice were then treated with antiPD-L1 monoclonal antibody alone or together with aspirin. (a) Tumor image, (b) tumor growth curve, and (c) tumor weight were shown. (d) IHC staining to check expression of Ki-67 and PD-L1. *N* = 5, ∗∗*P* < 0.01.

Next, we determined the effect of aspirin as an adjuvant for antiPD-L1 therapy on OC cell growth using an *in vivo* model. Tumorigenesis *in vivo* demonstrated that monoclonal antibody against PD-L1 attenuated the tumorigenesis of murine OC ID8 cells, whereas the addition of aspirin significantly enhanced the inhibitory effect of antiPD-L1 antibody, manifested by decreased tumor growth, tumor weight, and expression of Ki-67 and PD-L1 (Figures 6(a)–6(d)).

## 4. Discussion

Ovarian cancer is the primary reason for women malignancies-related mortality with poor prognosis worldwide. Immune checkpoint inhibitors that target PD-1/PD-L1 axis have been regarded as promising treatments in cancer treatment. Aspirin is a classic anti-inflammatory agent recently found to be highly potential and effective for treatments of multiple cancers. Nevertheless, the effect of aspirin on OC remains unclear. In this work, we determined to evaluate whether aspirin targeted PD-L1 signaling and OC development.

Studies have demonstrated the potential anticancer effect of aspirin on OC development. Aspirin inhibits EGF-stimulated viability of OC cells in a COX-1-dependent manner [18]. Aspirin promotes cisplatin sensitivity and represses tumor progression in epithelial OC [19]. Aspirin suppresses platelet-associated invasion of OC cells [20]. Moreover, a recent study reports that aspirin inhibits lung cancer cell growth by regulating the TAZ/PD-L1 signaling [21], presenting the effect of aspirin on PD-L1 in lung cancer. Our data indicated that treatment with aspirin suppressed the OC cell proliferation *in vitro*. Study on nude mouse model showed that aspirin treatment attenuated tumor growth and weight, along with decreased expression of Ki-67 and PD-L1. And the IFN-*γ*-stimulated PD-L1 expression in OC cells was suppressed by aspirin. Moreover, the PD-L1 and PD-1 expression of OC cells and activated T cells in the coculture system was inhibited under aspirin treatment. Aspirin treatment also recovered the proliferation and activation of T cells in the coculture system, as well as suppressed T cell apoptosis. Further study on *in vivo* mouse model proved the effect of antiPD-L1 therapy in OC. These results suggested the therapeutic effect of aspirin on OC development through targeting PD-1/PD-L1 signaling.

KAT5 is a lysine acetyltransferase that epigenetically modulates gene acetylation. Studies have reported the important function of KAT5 in cancer initiation and progression. For example, KAT5 stabilizes the expression of c-MYC to promote cell invasion and metastasis in ATC [22]. FosB recruits KAT5 to potentiate proliferation and metastasis of papillary thyroid cancer cells via modulating DPP4 function [23]. Besides, metformin inhibits melanoma development by repressing KAT5-regulated SMAD3 acetylation [24]. In our work, we demonstrated that KAT5 directly interacts with promoter of PD-L1 to recruit H3K27ac and RNA polymerase II and epigenetically modulate PD-L1 expression, whereas aspirin abolished this phenomenon by suppressing KAT5. These findings revealed the critical role of KAT5 in modulation of PD-1/PD-L1 signaling during OC development, suggesting the participation of KAT5 in OC immune therapy. Our data provided novel insight into the function of aspirin in OC treatment via modulating KAT5/PD-L1 signaling, suggesting the clinical significance of KAT5.

In conclusion, we discovered that aspirin suppressed PD-L1 expression through suppressing KAT5 and subsequently inhibited PD-1 and PD-L1 signaling to attenuate OC development. Aspirin may be an effective therapeutic drug for immunotherapy of OC.

## Data Availability

The datasets used and analyzed during the current study are available from the corresponding author on reasonable request.
